# A rare case of primary sinonasal tuberculosis presented with phlyctenular keratoconjunctivitis in a pediatric patient: A case report and literature review

**DOI:** 10.1097/MD.0000000000024787

**Published:** 2021-02-19

**Authors:** Thakoon Wiriyachai, Sophida Boonsathorn, Nopporn Apiwattanakul, Surapat Assawawiroonhakarn

**Affiliations:** a Department of Pediatrics, Faculty of Medicine Ramathibodi Hospital, Mahidol University, Bangkok; b Chakri Naruebodindra Medical Institute, Faculty of Medicine Ramathibodi Hospital, Mahidol University, Samut Prakan, Thailand.

**Keywords:** case report, phlyctenular kerotoconjunctivitis, primary sinonasal tuberculosis

## Abstract

**Rationale:**

Tuberculosis is a common cause of phlyctenular keratoconjunctivitis, especially for patients who live in a high endemic area of tuberculosis. We report a rare case of pediatric phlyctenular keratoconjunctivitis associated with primary sinonasal tuberculosis.

**Patient concerns:**

A 7-year-old boy presented with a 5-month history of redness of the left eye accompanied by mild visual impairment. Physical examination revealed elevated pinkish-white nodules with a circumcorneal hypervascularized lesion on the left conjunctiva.

**Diagnosis:**

Computed tomography revealed an enhancing soft tissue mass in the left maxillary sinus with bone destruction. Histopathology of maxillary tissue showed chronic inflammation without granuloma. Special stain, culture and polymerase chain reaction for mycobacterium were initially negative. Left maxillary sinus tuberculosis was diagnosed by positive *Mycobacterium tuberculosis* polymerase chain reaction from formalin-fixed paraffin-embedded maxillary tissue.

**Interventions:**

Two month of oral isoniazid, rifampicin, pyrazinamide, and ethambutol, followed by 10 months of oral isoniazid and rifampicin without topical eye drops agent were prescribed.

**Outcomes:**

Two months after initiation of treatment, the phlyctenular lesion had significantly improved. A follow-up computed tomography showed a significant reduction in the size of the maxillary sinus lesion and the extent of adjacent bone destruction.

**Lessons:**

Primary sinonasal tuberculosis is an uncommon cause of phlyctenular keratoconjunctivitis in children. When microbiological and histopathological evidences are absent, polymerase chain reaction analysis has a crucial role in the diagnosis of tuberculosis, especially in patient with uncommon presentation.

## 1 Introduction

Phlyctenular keratoconjunctivitis (PKC) has been described as a morphologic expression of a delayed-type hypersensitivity reaction to diverse antigens. It presents as inflamed nodules on the cornea and conjunctiva. *M. tuberculosis* is among the most common causes of PKC worldwide, particularly where tuberculosis (TB) remains endemic.^[1–3]^ Although extra-pulmonary manifestations of TB are commonly described in pediatric patients, sinonasal TB is extremely rare and could be misdiagnosed because of non-specific symptoms.^[4,5]^ We present a case of a 7-year-old boy diagnosed with primary sinonasal TB of the left maxillary sinus, presenting with PKC of the left eye.

## 2 Case presentation

**Figure 1:**
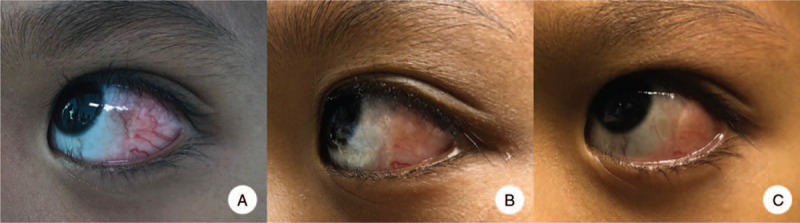
Phlyctenular lesion of the left eye upon admission (A), 1 month after initiation of treatment (B) and 2 months after initiation of treatment (C).

A previously healthy 7-year-old boy presented with a 5-month history of redness of the left eye accompanied by slight diminution of vision. He denied any history of fever, eye pain, abnormal eye discharge or other systemic symptoms, but noted a decreased vision. He had no history of TB exposure, respiratory illness, or previous trauma. Ophthalmological evaluation revealed elevated pinkish-white nodules with a circumcorneal hypervascularized lesion on the left conjunctiva measuring approximately 2 x 3 mm (Fig. 1). Full ocular motility was present in both eyes, pupils were round and equally reactive to light and accommodation reflexes were normal. The visual acuities of the right and left eye were 20/20 and 20/80, respectively. Examination of the fundus showed the right optic disc swelling with a choroidal fold. Rhinoscopy revealed bulging of the left uncinate process with congested inferior turbinates. Further examination was unremarkable. Initial laboratory findings included a white blood cell count of 10,710/μL with 60% neutrophils, a hemoglobin level of 10.2 g/dL, and a platelet count of 458,000/μL. Chest x-ray was unremarkable with no signs of hilar lymphadenopathy. Mantoux test was negative.

**Figure 2:**
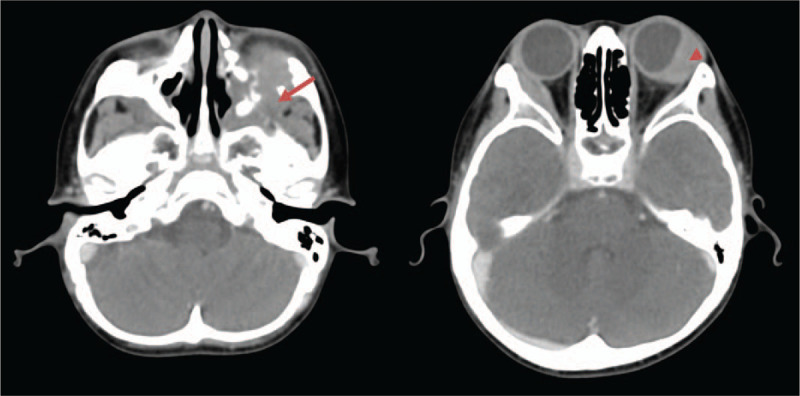
Computed tomography of the orbit demonstrated an enhancing soft tissue mass with extra-ocular muscle involvement of the left orbit (arrow head) and bone destruction in the left maxillary sinus (arrow).

Computed tomography (CT) showed an enhancing soft tissue mass, with extra-ocular muscle involvement and bone destruction in the left maxillary sinus and left orbit (Fig. 2). Rhabdomyosarcoma was first suspected. Histopathological examination of biopsy samples taken from subconjunctival nodules revealed chronic inflammatory tissue with histiocytic proliferation. Staining for microorganisms (including Gram stain, acid-fast stain, modified acid-fast stain) and polymerase chain reaction (PCR) for the detection of *M. tuberculosis* and non-tuberculous mycobacteria were unable to confirm the presence of any organisms.

The patient underwent antrostomy of the left middle meatus with tissue biopsy. Histopathology revealed benign respiratory mucosa with chronic inflammation without granuloma or malignant cells. Tissue biopsy of the lesion in the sub-Tenon's space revealed fibrous tissue with infiltration of small lymphocytes, histiocytes, and plasma cells, but no granuloma or malignant cells. Staining for acid-fast bacilli, modified acid-fast bacilli and Gömöri methenamine silver stain was negative. No organisms were isolated from aerobic cultures. Immunohistochemistry was suggestive of a reactive inflammatory infiltrate with numerous CD3- and CD20-positive lymphoid cells and CD68-positive histiocytic cells. Initially, PCR for *M. tuberculosis* from the maxillary sinus specimen was negative. However, because the ocular findings were suggestive of PKC and there was a strong suspicion of TB, formalin-fixed paraffin-embedded (FFPE) maxillary tissue was re-sent for *M. tuberculosis* PCR. This was positive for *M. tuberculosis* complex. A diagnosis of PKC with primary sinonasal TB of the left maxillary sinus was made, and anti-tuberculous therapy was initiated. This consisted of treatment with isoniazid, rifampicin, pyrazinamide, and ethambutol for 2 months. Mycobacterial culture did not identify the organism from all specimens. A follow-up CT of the orbit and sinus 6 weeks after initiation of treatment showed a significant reduction in the size of the infiltrative enhancing lesion and a decrease in the extent of bone destruction in the left orbit and maxillary sinus. Two months after initiation of this treatment, the patient's phlyctens had improved noticeably (Fig. 1). Isoniazid and rifampicin were continued to complete a 12-month course for tuberculous osteomyelitis of the maxilla.

## 3 Discussion

PKC is a cell-mediated hypersensitivity reaction of the cornea and/or conjunctivae. Common manifestations include excessive lacrimation, conjunctival injection, photosensitivity, blurred vision and the sensation of a foreign body in the eye. Progression to serious complications such as corneal ulceration and perforation or superimposed bacterial infection is possible if the condition is left untreated.^[2]^ In an area with a high prevalence of TB, PKC is usually associated with a variety of disease forms, including pulmonary TB, tuberculous lymphadenitis and spinal TB.^[1–3]^ Hypersensitivity to tuberculin protein may also be found, even without tubercular disease.^[2]^ Hence, patients presenting with PKC, particularly those who live in areas where TB is endemic, should be thoroughly investigated for the presence of TB infection.

In this case, following a finding of PKC, extensive investigations were performed to detect possible tuberculous diseases. Pulmonary TB and tuberculous lymphadenitis, 2 of the most common presentations of TB in children, were excluded. A CT scan revealed bone destruction involving the left maxillary sinus and left orbit, raising the suspicion of sinonasal TB. Primary sinonasal TB is relatively uncommon because of local protective mechanisms such as bactericidal secretion, ciliary movement, and mechanical filtering by nasal hairs.^[4,5]^ However, it was the most likely diagnosis in this case given the clinical picture and radiologic findings. Various diagnostic tests performed on maxillary tissue specimens, including histopathological examination, mycobacterial staining and culture, and PCR for *M. tuberculosis* complex, gave no supportive evidence for tuberculosis. Despite the lack of histopathological and microbiological evidence, primary left maxillary sinus TB was diagnosed from *M. tuberculosis* DNA detection in FFPE maxillary tissue using PCR. The literature suggests that PCR analysis for the diagnosis of TB from FFPE tissue gives 68.3% to 87.5% sensitivity and 95.1% to 100% specificity.^[6–8]^ Therefore, this technique is useful for the diagnosis of TB in cases where, despite strong clinically suspicion, histopathologic findings are not confirmatory.^[9–11]^ In this case, recognition of the distinctive ophthalmic findings associated with TB, along with the use of PCR, meant that sinonasal TB could be diagnosed.

Amongst pediatric PKC cases, pulmonary TB is the most commonly associated tuberculous disease.^[1–3,12]^ According to recent publications, the most common extrapulmonary TB associated with PKC in children is tuberculous lymphadenitis.^[12–18]^ To the best of the authors’ knowledge, this is the first report of a pediatric PKC case associated with definite primary sinonasal TB, confirmed by molecular diagnosis (Table 1).

**Table 1:** Publications of pediatric phlyctenular keratoconjunctivitis cases associated with tuberculosis since 2000.

Publications	Patients	Primary site of TB	Diagnostic test for TB	Management
Rohatgi J^[12]^ 2000	N = 56,3–38 yrs old	Pulmonary TBTB lymphadenitis	- CXR findings compatible with pulmonary TB- Pathological examination compatible with TB	Oral anti-TB agents with steroid eye drops
Singal A^[13]^2006	i. 6-yr-old boyii. 5-yr-old girl	Cutaneous TB: Lupus vulgarisCutaneous TB: Scrofulosorum	- Skin Bx: granuloma with positive AFB stain- Skin Bx: caseous granuloma- TB culture (skin): *M* tuberculosis complex	Oral anti-TB agents with steroid eye dropsOral anti-TB agents with steroid eye drops
Sharma K^[14]^2014	6-yr-old girl	TB lymphadenitis	- Mantoux test: positive- Lymph node Bx: central necrosis with multinucleated giant cell with positive AFB stain	Oral anti-TB agents with steroid eye drops
Lahiri K^[15]^2015	4-yr-old girl	Pulmonary TB	- Mantoux test: positive- CXR findings compatible with pulmonary TB	Oral anti-TB agents
Tomar M^[16]^2016	2.5-yr-old boy	Pulmonary TB	- Mantoux test: positive- CXR findings compatible with pulmonary TB	Oral anti-TB agents
Rahman MH^[17]^2018	8-yr-old girl	TB lymphadenitis	- Lymph node Bx: caseation necrosis with giant cell- TB PCR (lymph node): positive	Oral anti-TB agents
Balyan M^[18]^2019	11-yr-old girl	Pulmonary TB	- Mantoux test: positive- CXR findings compatible with pulmonary TB	Oral anti-TB agents with steroid eye drops
This case	7-yr-old boy	Sinonasal TB	- Maxillary sinus tissue Bx: chronic inflammation without granuloma or malignant cells- TB PCR (maxillary sinus tissue): positive	Oral anti-TB agents

The recommended management of PKC associated with TB usually consists of systemic anti-tuberculous agents and topical steroids applied to the affected eye.^[2]^ Our patient received 12 months of systemic anti-tuberculous therapy regimen without topical treatment and responded well without complications.

## 4 Conclusion

We describe a rare case of PKC associated with primary maxillary sinonasal TB, one of the distinctive forms of tuberculous disease associated with PKC. PCR analysis plays an important role in the diagnosis of TB, especially when microbiological and histopathological findings are absent.

## Acknowledgments

We thank Andrew Bailey from Edanz Group (https://en-author-services.edanzgroup.com/ac) for editing a draft of this manuscript.

## Author contributions

**Conceptualization and design:** Sophida Boonsathorn, Surapat Assawawiroonhakarn

**Data curation:** Thakoon Wiriyachai, Sophida Boonsathorn

**Investigation:** Thakoon Wiriyachai, Sophida Boonsathorn

**Methodology:** Surapat Assawawiroonhakarn, Sophida Boonsathorn.

**Supervision:** Nopporn Apiwattanakul, Sophida Boonsathorn, Surapat Assawawiroonhakarn

**Writing – original draft:** Thakoon Wiriyachai, Surapat Assawawiroonhakarn

**Writing – review & editing:** Thakoon Wiriyachai, Surapat Assawawiroonhakarn, Sophida Boonsathorn, Nopporn Apiwattanakul. All authors contributed to the review, editing, and approval of the final manuscript.
